# Evaluation of *Trichoderma atroviride* and *Trichoderma longibrachiatum* as biocontrol agents in controlling red pepper anthracnose in Korea

**DOI:** 10.3389/fpls.2023.1201875

**Published:** 2023-07-14

**Authors:** Seung Hwan Kim, Younmi Lee, Kotnala Balaraju, Yongho Jeon

**Affiliations:** ^1^ Department of Plant Medicals, Andong National University, Andong, Republic of Korea; ^2^ Agricultural Science & Technology Research Institute, Andong National University, Andong, Republic of Korea

**Keywords:** *Trichoderma* spp., biological control, *Colletotrichum acutatum*, VOCs, disease suppression

## Abstract

Anthracnose disease is a serious threat to red pepper crops in Korea and many other countries, resulting in considerable yield losses. There are now no effective control techniques available except for fungicide sprays, which may directly impact consumers. This study aims to investigate the biological activity of *Trichoderma* isolates in controlling red pepper anthracnose caused by *Colletotrichum acutatum in vitro* and in the field. Out of 11 *Trichoderma* isolates screened for biocontrol agents against three fungal pathogens, including *C. acutatum*; two effective *Trichoderma* isolates, *T. atroviride* ATR697 (ATR697) and *T. longibrachiatum* LON701 (LON701) were selected for further investigation. Using the overlapping plates experiment, it was discovered that the volatile organic compounds (VOCs) produced by ATR697 strongly inhibited *C. acutatum* mycelial growth to a larger extent than the isolate LON701. A cellophane membrane experiment has shown that mycelial growth of *C. acutatum* was inhibited by 36% and 27% when treated with ATR697 and LON701, respectively. Culture filtrates (CFs) of two *Trichoderma* isolates inhibited the mycelial growth of *C. acutatum in vitro*. When red peppers were treated with spore suspensions of LON701 and ATR697, the disease severity (%) was 44.1% and 55.8%, respectively, in a curative method; while the disease severity (%) was 5% and 11.6%, in LON701- and ATR697-treated red peppers, respectively, in a preventive method. These results showed the suppression of disease severity (%) was relatively higher in the preventive method than in the curative method. Furthermore, *Trichoderma* isolates ATR697 and LON701 were resistant to commercial chemical fungicides *in vitro*, indicating these strains may also be used synergistically with a chemical fungicide (pyraclostrobin) against the growth of *C. acutatum*. There was no difference in the inhibition rate (%) of the pathogen between the treatment with LON701 alone and LON701+pyraclostrobin. Based on *in vitro* findings, ATR697 and LON701 played a role in effectively controlling red pepper anthracnose in field conditions, with LON701 treatment resulting in a disease rate of 14% when compared to ATR697, chemical, and non-treated controls. Overall, our study showed the ability of *Trichoderma* isolates to control red pepper anthracnose and their potential to develop as novel biocontrol agents to replace chemical fungicides for eco-friendly, sustainable agriculture.

## Introduction

Pepper (*Capsicum annum* L.) is one of the most important vegetable crops in the world, including Korea; however, its yield and quality have been frequently limited by various diseases, including anthracnose, caused by *Colletotrichum* spp. (Lee et al., 2012). One of the most common fungal infections in pepper is *Colletotrichum* species complex, which causes considerable fruit losses during pre- and post-harvest conditions (Baba et al., 2019). Anthracnose disease also causes damage to several crops, including mangoes (Li et al., 2020), bananas (Zakaria et al., 2009), apples (Kim et al., 2016), and tomatoes (Diao et al., 2014). *Colletotrichum* species have been shown to infect plants by spore germination, appressoria development, and hyphal penetration in the host epidermis (Yan et al., 2018). Disease symptoms were found to increase during the rainy season because conidia of *Colletotrichum* are disseminated onto fresh fruit, resulting in a secondary infection (Suparman et al., 2017). The disease symptoms differ depending on the plant part affected; it causes damage in fruits in particular, appearing as dark, necrotic lesions with concentric acervuli rings, leading to pre- and post-harvest rot (Saxena et al., 2016).

Application synthetic fungicides is still practiced for controlling anthracnose diseases in Korea, and many synthetic fungicides, such as pyraclostrobin, tebuconazol, hexaconazole, propineb, and thiophanate-methyl are used for the control of anthracnose disease (Gao et al., 2021; Kim et al., 2021); however, the chemical method is not always effective to control the disease incidence. The continuous application of synthetic fungicides causes toxicity and side effects on humans and the environment, resulting in public concern and the need to search for eco-friendly methods to limit the adverse impact on human beings and the environment as well (Fang et al., 2022). Therefore, an alternative method is needed to address this issue, and safer agricultural policy necessitates the employment of biological control agents (BCAs) to control plant diseases. Among these, fungi classified under the genus *Trichoderma* are dominant, commonly found in soil ecosystems, and grow along root surfaces and immediately below the outermost cells of the roots (Błaszczyk et al., 2014; Tyskiewicz et al., 2022). *Trichoderma* feed on soil microbes surrounded by rhizosphere by root exudates (Rouina et al., 2021). They are saprophytic microorganisms with a comprehensive metabolism that is capable of utilizing a variety of substrates (Mukhopadhyay and Kumar, 2020).

*Trichoderma* species have been extensively studied for their applications in agriculture due to their well-known biological control mechanisms (Zin and Badaluddin, 2020), and bioremediation (Sharma et al., 2016). *Trichoderma* can suppress the growth of plant pathogens by several means, such as mycoparasitism, antibiosis, encounter for nutrients uptake and space, and effective host resistance (Zhang et al., 2022). *Trichoderma* can be found in a variety of ecosystems, including rhizosphere soil (Zin and Badaluddin, 2020) and plants (Cummings et al., 2016) that positively affects plant growth and defense (Geng et al., 2022). Various species of *Trichoderma* are identified as BCAs due to their potential to control a wide range of plant diseases (Rebolledo-Prudencioa et al., 2020). *Trichoderma* inoculants can be used to help plants overcome environmental stresses to reach their yield potential (Abirami et al., 2022), this is possibly by root colonization which can increase seedling vigor and plant immune system (Agostini et al., 2023), and act as a plant antioxidant, increasing photosynthesis efficiency in stressed plants (Oljira et al., 2020). *Trichoderma* has been reported as an essential endophyte that can interact with various plants (Zhang et al., 2014; Lamdan et al., 2015), which gives a significant advantage to the agriculture industry to prevent the deposition of synthetic chemical residues. Thus, using *Trichoderma* species as BCAs seems to be a notable approach for sustainable agriculture.

Although several reports have shown the application of *Trichoderma* in controlling plant diseases in several crops, such as apples (Zhang et al., 2022), tomatoes (Olowe et al., 2022), bananas (da Costa et al., 2021), and pepper (Ezziyyani et al., 2007), there is no report on the biocontrol efficacy of *Trichoderma* isolates against red pepper anthracnose in Korea. Therefore, the objectives of this study were to (i) isolate *Trichoderma* strains from soil samples collected from the mountain regions; (ii) evaluate their antagonistic activity against fungal pathogens *in vitro*; (iii) determine the effect of volatile and non-volatile metabolites from *Trichoderma* isolates on mycelial growth of the fungal pathogen *C. acutatum in vitro*; and (iv) determine the biocontrol activity against red pepper anthracnose under *ex vivo* and field conditions.

## Materials and methods

### Isolation of *Trichoderma* species from soil samples

Soil samples were collected from four different geographical locations, in Andong, Gyeongsangbuk Province, Korea, i.e., Ongcheon-ri (36°41’35.5”N 128°41’39.8”E), Mulhan-ri (36°40’12.0”N 128°42’34.0”E), Namseon myeon (36°32’06.0”N 128°47’04.6”E), and Seonyeon-ri (36°40’55.6”N 128°44’52.8”E) of apple orchards near mountain regions. The soil sample was collected 50 cm away from the apple tree and 5 cm below the ground. One gram of soil was taken in a Falcon tube (50 mL) containing sterile distilled water (SDW) and shaken (180 rpm) for 1 h. The samples were diluted five times, and 100 µL was spread onto potato dextrose agar (PDA) plates. Fungal colonies were collected 7 d after incubation at 25°C. The internal transcribed spacer (ITS) region served as the basis for the selection and identification of 40 putative colonies.

### Molecular identification of *Trichoderma* isolates

For molecular identification of the antagonistic *Trichoderma* spp., PCR for the amplification of the ITS region was performed (El-Sobky et al., 2019). Genomic DNA from *Trichoderma* isolates was extracted using the Genomic DNA Prep Kit (Biofact Co., Seoul, Korea) following the manufacturer’s instructions. The isolated genomic DNA was stored at –80°C until further use. The ITS region was amplified using the primer pair ITS1 (5’-TCCGTAGGTGAACCTGCGG-3’) and ITS4 (5’-TCCTCCGCTTATTGATATGC-3’) (White et al., 1990). The PCR was performed in a total reaction volume of 50 μL, containing 50 ng of template DNA (2 μL), 0.5 mM of forward primer (2 μL), 0.5 mM of reverse primer (2 μL), 1.25 U *Taq* DNA polymerase (0.25 μL) (Solgent, Daejeon, South Korea), 5 μL 10X *Taq* buffer, and 32.25 μL SDW. It took 35 cycles of extension at 72°C for 1 min, extension at 72°C for 5 min, and cooled at 4°C to complete the PCR amplification of ITS. Initial denaturation at 95°C for 1 min, denaturation at 95°C for 20 s, and annealing at 52°C for 40 s. The PCR products were purified using a PCR purification kit (Biofact Co., Seoul, Korea). The PCR product obtained was sequenced by an automated sequencer (Genetic Analyzer 3130; Applied Biosystems, Carlsbad, CA, USA) at Solgent, Daejeon, South Korea. The same primers as above were used for this step. The analyzed DNA nucleotide sequences were compared with existing DNA nucleotide sequences using the NCBI-BLAST tool. Sequence alignment and phylogenetic tree construction were performed using the DNASTAR software (Version 5.02, DNA Star Inc., Madison, USA) and MEGA 4.0, respectively (Biodesign Institute, Tempe, USA).

### Fungal pathogen cultures

Pathogenic fungal cultures including *Fusarium oxysporum* KACC40043, and *Phytophthora capsici* KACC40473 were obtained from the Korean Agricultural Culture Collection (KACC), NAAC, RDA, Korea; and the pathogen *Colletotrichum acutatum* GYUN-10586 used in this study was from our laboratory’s collection. This pathogen was isolated according to the modified method followed by Lin et al. (2021). In brief, the symptomatic red pepper fruit tissue (0.5 cm^2^) was surface sterilized with 1% sodium hypochlorite (NaClO) for 1 min, washed with SDW twice, plated onto PDA plates, and incubated under 12-h/12-h cycles of light and darkness at 25°C for 4 d. To obtain pure colonies, the fungal colonies were transferred to freshly prepared PDA plates. The cells were cultured on PDA plates and stored at 4°C until further use. Conidial suspensions were prepared by suspending mycelia scraped from 7-day-old cultures of pathogenic fungus culture on a PDA plate. The resulting suspensions were filtered through a double-layered cheesecloth. Before application, the spore suspensions were adjusted to 10^5^ conidia/mL using a hemocytometer. For long-term preservation, spore suspensions of fungal pathogens were maintained at –80°C in potato dextrose broth (PDB) with glycerol (20%).

### Dual culture plate assay

A total of 11 *Trichoderma* species (*T. atroviride, T. asperellum, T. harzianum, T. hamatum, T. koningiopsis, T. lixii, T. longibrachiatum, T. reesei, T. tomentosum, T. virens*, and *Trichoderma* sp.) were selected based on ITS region and screened for antagonistic activity against mycelial growths of *C. acutatum* (anthracnose), *F. oxysporum* (wilt), and *P. capsici* (blight), using a dual culture plate assay as per the procedure developed by Castillo et al. (2011). An agar plug from a 5-day-old fungal pathogen colony was placed on one side of a Petri dish (9 cm diameter), with an agar plug of each *Trichoderma* sp. placed on the opposite side, 5 cm distant from the pathogen. PDA plates inoculated with the pathogen alone were used as the controls. The experiment used a complete randomized block design (CRBD) and was performed twice with five replicates. The pathogen’s radial growth was assessed after 7 d of incubation at 25°C, and the percentage inhibition was computed using the method provided by Rahman et al. (2009), using the following equation:

Percentage inhibition (%) = R1−R2/R1 × 100, where R1 and R2 are radial growths of pathogen in the control and treatment, respectively.

### Morphological characterization of isolates ATR697 and LON701

Based on conidial characteristics described by Phillips et al. (2013), morphological characterization of the fungal mycelia was examined. After mycelia were fully grown on PDA plates, the formation of microstructures such as conidiophores and conidia was induced by incubation at 28°C, 12/12 photoperiod for 3 weeks. When the colonies had nearly completely covered the plate, the entire plate, the diameters of the colonies were measured along two perpendicular axes, and the data was translated to daily radial growth (mm per day). For microscopy analysis, fungal hyaline hyphae were cut and placed in 100% lactic acid, and morphological characters of the conidia and conidiogenesis were observed under a ProgRes speedXT ^core^3 Imager microscope. These images were captured with an AxioCam MRc5 camera (Zeiss, Germany).

### Fungal pathogen−*Trichoderma* volatile−exposure bioassay using the overlapping plates assay

To determine the effect of the volatile organic compounds (VOCs) secreted by *Trichoderma* sp., against the growth of fungal pathogens, exposure of *Trichoderma* VOCs was performed using the overlapping plates assay. Two *Trichoderma* isolates, namely *T. atroviride* ATR696 and *T. longibrachiatum* LON701, were cultured on PDA plates at 25°C for 7 d. Mycelial discs of *Trichoderma* were cut using a sterile cork borer (5 mm diameter) and placed at the center of a freshly prepared PDA plate. A mycelial disc (5 mm diameter) of the fungal pathogen *C. acutatum* GYUN-10586 was placed onto another freshly prepared PDA plate in the same manner. PDA plates inoculated with *C. acutatum* mycelial plugs were placed on top of the PDA plates inoculated with *Trichoderma* species and the plates were then sealed with parafilm. The inhibition of mycelial growth of *C. acutatum* was observed after incubating the plates at 25°C for 14 d. The experiment was performed twice in triplicates (Petri dishes).

### Cellophane membrane experiment

To determine the effects of antifungal non-volatile metabolites secreted by the *Trichoderma* species, a cellophane membrane experiment was performed using the method described by Li et al. (2018). A plug from 7-day-old *Trichoderma* isolates, ATR696 and LON701 cultured on a PDA plate was inoculated on a cellophane membrane (LABISKOMA Ltd., Seoul, Korea) overlaid onto PDA plates. The cellophane membrane with *Trichoderma* culture was removed after 3 d of incubation at 25°C, and a plug of *C. acutatum* culture was inoculated on the same medium to measure the level of fungal mycelia growth inhibition. The experiment was performed twice in triplicates (Petri dishes).

### Inhibition effect of culture filtrate from *Trichoderma* isolates

The effect of culture filtrates (CF) of *Trichoderma* isolates against the growth of the fungal pathogen *C. acutatum* under *in vitro* conditions was determined as follows. The two *Trichoderma* isolates, such as ATR697 and LON701, were cultured in a 500 mL Erlenmeyer flask containing 200 mL of PDB or M9 broth at 25°C under shaking conditions (150 rpm). Six days after incubation, 20 mL was taken and centrifuged at 3000 × *g* and 4°C for 20 min and filtered using a syringe and 0.22 µm filter using an aseptic technique. Therefore, 200 µL of the CF was spread onto PDA plates, and a 7-day-old cultured *C. acutatum* mycelium plug was placed onto a PDA plate. Plates treated with PDB alone served as a non-treated control. All the plates were incubated in the dark at 28°C for 7 d. Mycelial growth (mm in diameter) inhibition rate (%) was recorded after incubating the plates at 28°C for 7 d. The experiment was performed twice in triplicates.

### *Ex vivo* assay to test *Trichoderma* isolates against red pepper anthracnose

An *ex vivo* experiment was conducted to examine the preventive and curative effects of *Trichoderma* isolates ATR697 and LON701 on anthracnose suppression in wounded red pepper fruits (cv. green light). Fully ripened red peppers were selected for the experiments. The fruits were surface-sterilized in 70% ethanol for 3 min, submerged in 1% NaOCl for 1 min, and then rinsed 2–3 times in SDW. The red pepper fruits were then air-dried at room temperature. Wounds were created on the surface of the fruits using a sterilized needle. To test for preventive effects, the wounded fruits were treated with antagonistic *Trichoderma* suspensions of ATR697 and LON701 at different concentrations (10^7^, 10^6^, C10^5^, and 10^4^ spores/mL) by spot inoculating the wounded site with a micropipette. To prepare the suspensions, the two *Trichoderma* isolates were individually cultured on PDA plates. Seven days after incubating the plates at 25°C, the spore suspensions were washed with SDW before filtering with double-layered gauze. The concentration was estimated using a hemocytometer and adjusted appropriately. After drying at room temperature, 10 μL of *C. acutatum* spore suspension (10^5^ conidia/mL) was inoculated on the wounded sites. They were then placed on square plates (40 × 40 cm) containing moist filter paper to maintain humidity. Fruits treated with pyraclostrobin and SDW served as positive and negative controls, respectively. The disease index was measured 10 d after incubation at 25°C and compared to a non-treated control; the control value (%) was computed from this. Wounded red pepper fruits were inoculated with 10 μL of *C. acutatum* spore suspension (10^5^ conidia/mL), dried at room temperature, and placed on square plates (40 × 40 cm) with moist paper to maintain humidity. After incubating the plates at 25°C for 1 d, the wounded sites of the fruits were treated with 10 μL of spore suspensions of two antagonistic *Trichoderma* isolates ATR697 and LON701 at various concentrations (10^7^, 10^6^, C10^5^, and 10^4^ spores/mL), and incubated at 25°C for another 7 d. Fruits treated with pyraclostrobin and SDW served as the positive and negative controls, respectively. The disease severity (%) was calculated as follows: A disease index scale was established based on the score from 0 to 5 (0 = no symptoms, 1 = lesions with<10% disease incidence, 2 = symptoms with 11–20%, 3 = 21–40%, 4 = 41–70%, and 5 = symptoms with >71-100%. Disease severity (%) = [sum (class frequency × score of rating class)]/[(total number of fruits) × (maximal disease index)] × 100.

### Fungicide sensitivity test against antagonistic *Trichoderma* isolates

To investigate the fungicidal sensitivity of the two *Trichoderma* isolates, ATR697 and LON701, three chemical fungicides (pyraclostrobin, tebuconazol, and kresoximmethyl) that are currently used to control red pepper anthracnose in Korea were used. These chemicals (0.2%) were dissolved in SDW and mixed with autoclaved PDA (cooled to approximately 50°C). Before the medium solidified, the medium containing the fungicide was added to Petri dishes. The control plates contained PDA without any fungicides. A mycelium plug (5 mm diameter) of two *Trichoderma* isolates cultured for 7 d was placed on the center of the PDA plates with or without fungicides, and the plates were incubated at 25°C for 7 d. Mycelia growth was observed, and results were compared to mycelia grown on PDA plates without fungicides. The diameter of each colony of the microorganism was measured once the fungicide-free control plates’ colonies had covered 75% of the agar surface. *Trichoderma* resistance and sensitivity to fungicides were determined on the basis of the inhibition zone. The experiment was carried out twice with three replicates for each treatment. The mycelial growth rate inhibition was determined as a percentage (%).

### Disease control of red pepper anthracnose by the synergistic effects of *Trichoderma* isolates and chemical treatment

Wounded red pepper fruits were treated with a combined application of a chemical (20% WG pyraclostrobin) and *Trichoderma* isolates ATR697 and LON701 at two different concentrations (10^5^ and 10^4^ spores/mL) using the preventive method described above. The chemical (pyraclostrobin) was dissolved in SDW (0.335mg/mL) and from this; 10 µL was placed on the wounded site of the red pepper fruit, an hour after air-dried, 10 µL suspensions (10^5^ and 10^4^ spores/mL) of *Trichoderma* isolates were placed on the same site. After 24 h of incubation at 25°C, the treated red pepper fruits were inoculated with a 10 μL of fungal pathogen suspensions (10^5^ spores/mL) of *C. acutatum*, and fruits were kept in square plates (40 × 40 cm) containing moist paper to maintain humidity, were incubated at 25°C for 7 d. The disease index representing the disease severity (%) was then recorded. Fruits treated with pyraclostrobin and SDW served as the positive and negative controls, respectively. Each experiment was performed twice, with ten replicates (fruits) per treatment.

### Field evaluation of the effects of *Trichoderma* isolates on disease suppression of red pepper anthracnose

Two *Trichoderma* isolates, ATR697 and LON701, were used in a field experiment to investigate the biological control of red pepper anthracnose at Andong National University, Andong, Korea. Three-week-old red pepper seedlings were transplanted in a field with 100 cm between rows and 40 cm between the individual plants. Non-woven fabric was used to cover planting rows in order to prevent weed growth. The selected field was a naturally disease-infested field with red pepper anthracnose, caused by *C. acutatum*. The treatments with antagonistic *Trichoderma* isolates were categorized into two groups, namely, foliar spray and foliar spray + soil drenching. The experiment was conducted in two consecutive years, 2021 and 2022. *Trichoderma* suspensions prepared at a concentration of 10^7^ spores/mL were used for all the treatments. The first dose was administered on July 2^nd^ and June 25^th^, in 2021 and 2022, respectively. Six treatments were administered at 10 d intervals in both years until August 31. Pyraclostrobin emulsion and tap water were used as positive and negative controls, respectively. Disease-infected fruits were collected 10 d after the last treatment. Disease severity (%) was calculated using a disease index. The disease severity (%) of the treatments was compared with that of non-treated and chemical controls. Each treatment consisted of 20 replicates (red pepper plants). The disease rate (%) was calculated using the following formula:


Disease rate(%)=(Total fruits–healthy fruits)/total fruits×100

### Statistical analysis

Data were analyzed using the R software [(RStudio “Prairie Trillium” Release (2022-05-20)] for Windows. The independence test was used based on Pearson’s chi-squared test (Agresti, 2007). One-way analysis of variance (ANOVA) was used (Chambers et al., 1992). Means were separated using the least significant difference test (LSD) at *P<* 0.05.

## Results

### Isolation of *Trichoderma* species and *in vitro* antagonistic screening against fungal pathogens

**Figure 1:**
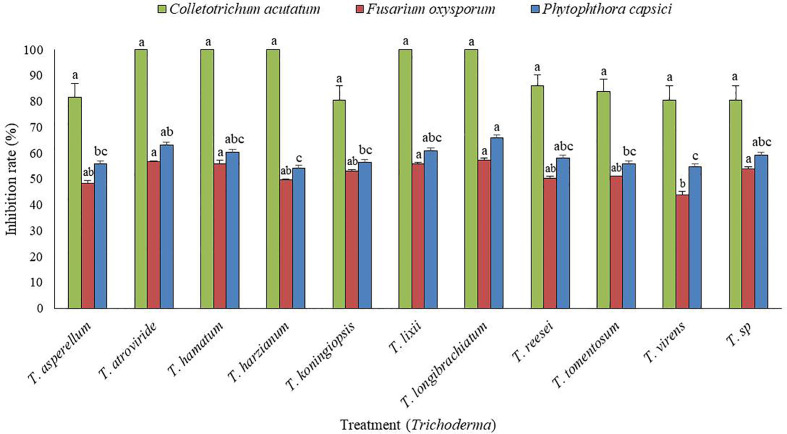
*In vitro* screening of antagonistic activity of 11 *Trichoderma* isolates against mycelial growths of three fungal pathogens (*Fusarium oxysporum*, *Phytophthora capsici*, and *Colletotrichum acutatum*) by dual culture plate assay. The inhibition zone was measured 7 d after incubating at 25°C. The experiment was performed at least twice with five replications per treatment. Bars indicate the standard error of the mean, and bars with the same letters do not differ from each other according to the least significant difference (*P*< 0.05).

**Figure 2:**
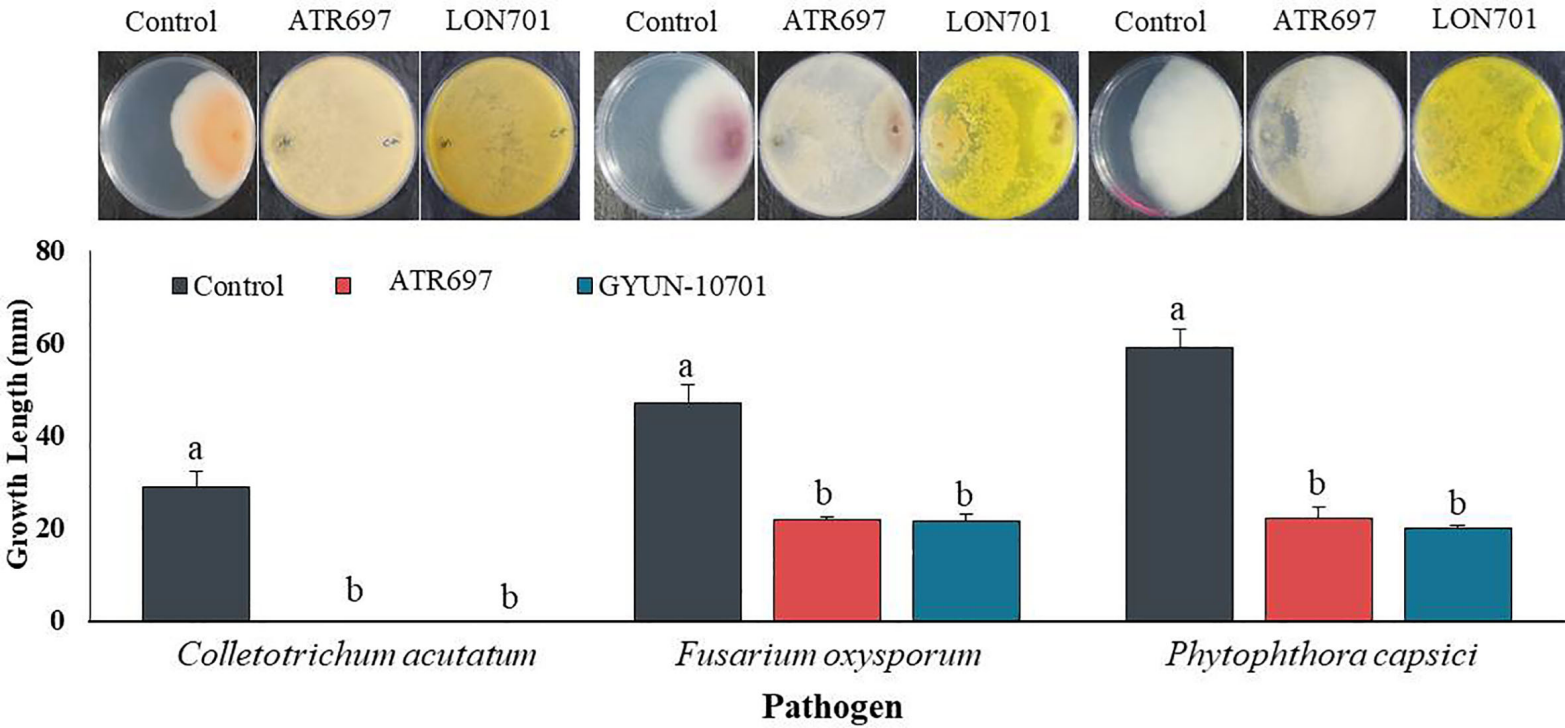
Inhibition of mycelial growth of three fungal plant pathogens by treatment with the two *Trichoderma* isolates ATR697 and LON701 using a dual culture plate assay under *in vitro* conditions. The inhibition percentage was recorded 7 d after incubating the plates at 25°C. The experiment was performed twice with three replicates per treatment. Bars with the same letters do not differ from each other according to the least significant difference (LSD) at *P<* 0.05. *C.a*: *Colletotrichum acutatum*, *F.o*: *Fusarium oxysporum*, and *P.c*: *Phytophthora capsici.*.

From a total of 40 fungal isolates obtained from the soil sample, only 11 isolates have been identified as *Trichoderma* species. Of which, only two *Trichoderma* isolates, such as *T. atroviride* ATR697 (ATR697) and *T. longibrachiatum* LON701 (LON701) were selected based on *in vitro* antagonistic activity against three fungal pathogens, *C. acutatum*, *F. oxysporum*, and *P. capscici* (**Figure 1**). The two *Trichoderma* isolates, ATR697 and LON701 were found to inhibit the mycelial growths of the three above said fungal pathogens. These two *Trichoderma* isolates have the highest *in vitro* antagonistic activity against *C. acutatum*, with a 100% inhibition value. Whereas, the growth inhibitions of *F. oxysporum* and *P. capsici* were displayed as 56.7% and 63.3%, respectively, by treatment with ATR697. Meanwhile, in treatment with LON701, the mycelial growth inhibition rates of *F. oxysporum* and *P. capsici* were 57.4% and 66.1%, respectively (**Figure 2**).

### Molecular identification of *Trichoderma* isolates

**Figure 3:**
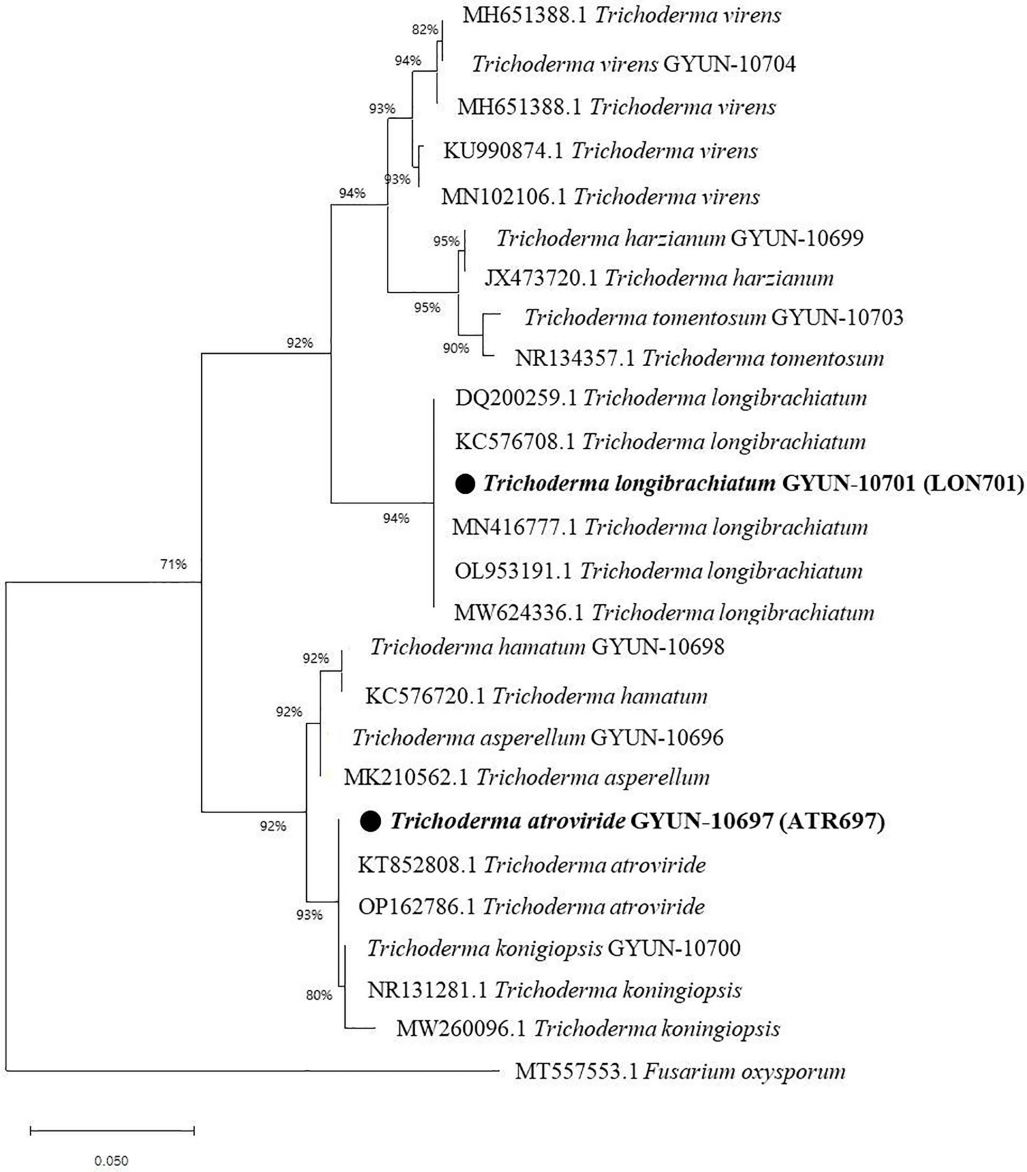
Phylogenetic dendrogram constructed from a comparative analysis of ITS gene sequences showing the relationships between *T. atroviride* ATR697 and related *Trichoderma* species, and relationships between *T. longibrachiatum* LON701 and related *Trichoderma* species. Bootstrap values (expressed as percentages of 1000 replications) > 50% are shown at branch points and the species names are followed by the GenBank accession numbers. The maximum parsimony phylogenetic tree was generated by the MEGA 4.0 program. The scale bar indicates 0.02 substitutions per nucleotide position.

The two *Trichoderma* isolates were identified by PCR using an internal transcribed spacer (ITS)-specific primer sequence. A total of 11 *Trichoderma* isolates were identified, including *T. asperellum, T. atroviride, T. hamatum, T. harzianum, T. koningiopsis, T. lixii, T. longibrachiatum, T. reesei, T. tomentosum, T. virens*, and *T.* sp. (**Table 1**), of which two effective antagonist strains (ATR697 and LON701) were subjected to phylogenetic analysis. Based on this, a phylogenetic tree was constructed using ITS sequences from *Trichoderma* isolates with sequences retrieved from GenBank. Gene sequences of both isolates ATR697 and LON701 were deposited in NCBI with GenBank accession numbers OQ772187 and OQ772191, respectively. BLAST analysis yielded 99.8%, 99.8%, and 99.8% identity with *T. atroviride* strains (accession no. ON203997.1, KT852808.1, and OP162786.1); while 100%, 100%, and 100% identity with *T. longibrachiatum* strains (accession no. MN416777.1, OP938775.1, and DQ200259.1). Phylogenetic analysis of these isolates clustered with other *Trichoderma* spp. The isolates were confirmed to be *Trichoderma atroviride* ATR697 and *Trichoderma longibrachiatum* LON701 (**Figure 3**).

**Table 1:** Identification of *Trichoderma* species.

Trichoderma isolate	strain	Qurey cover (%)	Percent Ident (%)	Source
*T. asperellum*	GYUN-10696	99	99.83	soil
*T. atroviride*	ATR697	99	99.84	soil
*T. hamatum*	GYUN-10698	100	100	soil
*T. harzianum*	GYUN-10699	100	99.36	soil
*T. koningiopsis*	GYUN-10700	100	99.18	soil
*T. lixii*	GYUN-10694	100	99.52	soil
*T. longibrachiatum*	LON701	100	100	soil
*T. reesei*	GYUN-10702	99	99.05	soil
*T. tomentosum*	GYUN-10703	100	99.36	soil
*T. virens*	GYUN-10704	99	99.68	soil
*T.* sp	GYUN-10705	100	99.68	soil

### Morphological characteristics of strains ATR697 and LON701

**Figure 4:**
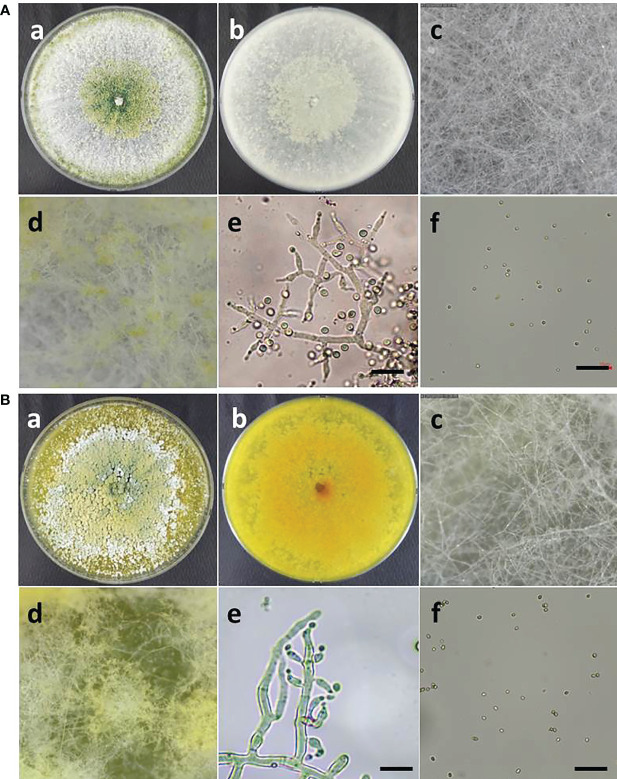
**(A)** Morphological characteristics and microscopic observations of *Trichoderma atroviride* ATR697. Colony morphology of ATR697 on PDA plate on the front side **(a)** and the reverse side **(b)**. Growth of mycelia on 2^nd^ day **(c)** and 4^th^ day **(d)** on PDA plate. Fungal hyphae and spores **(e)**, and conidia **(f)** of ATR697, measures 3.0-4.0 × 2.0-3.0 (length × width) µm. Scale bars = 10 µm. **(b)** Morphological characteristics and microscopic observations of *Trichoderma longibrachiatum* LON701. Colony morphology of LON701 on PDA plate front side **(a)** and the reverse side **(b)**. Growth of mycelia on 2^nd^ day **(c)** and 4^th^ day **(d)** on PDA plate. The hyphae and spores **(e)**, and conidia **(f)** of LON701, measures 4.0-5.0 × 2.0-3.0 (length × width) µm. Scale bars = 10 µm.

The morphological characteristics of two *Trichoderma* species, such as *T. atroviride* ATR697 and *T. longibrachiatum* LON701, were observed on the PDA medium. Conidia of each strain were observed under a microscope (**Figure 4**). The mycelia of *T. atroviride* ATR697 appeared as greenish at the center and edges of the plate as well; it appears in light grey on the reverse side of the plate (**Figure 4A**). The conidia were round and measured 3.0-4.0 × 2.0-3.0 (length × width) µm. Whereas the growth of mycelia of *T. longibrachiatum* LON701 appears light yellow on the front side of the PDA plate, but it appears yellow color on its reverse side (**Figure 4B**). The conidia were round shaped and measured 4.0-5.0 × 2.0-3.0 (length × width) µm.

### Effect of volatile organic compounds (VOCs) of *Trichoderma* isolates on mycelial growth of pathogenic fungus *C. acutatum* under *in vitro* conditions

**Figure 5:**
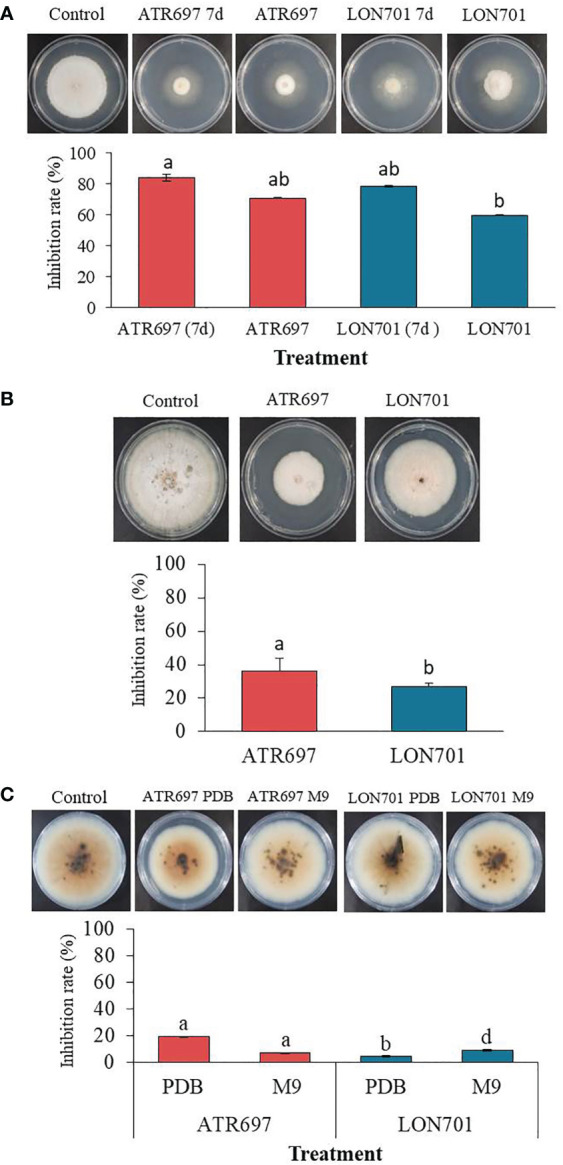
**(A)** The inhibitory effect of volatile organic compounds (VOCs) produced by *Trichoderma* isolates, ATR697 and LON701 against the growth of fungal pathogen *C acutatum* was tested using the overlapping plates assay. One set of plates inoculated with *Trichoderma* isolates and cultured for 7 d were overlapped with a pathogen-inoculated plate, and another set of plates both *Trichoderma* and pathogen inoculated on the same day were overlapped. The colony diameter was measured at 14 d after incubating *C acutatum* plates at 25°C. **(B)** Effect of secondary metabolites by *Trichoderma* isolates on the growth of pathogenic fungal mycelia using a cellophane membrane. *Trichoderma* isolates cultured on the cellophane membrane overlaid on PDA plates. A plug of fungal pathogen *C acutatum* was inoculated 3 d after removing cellophane membrane. The growth inhibition was measured 14 d after incubating at 25°C. **(C)** The inhibitory effect of culture filtrate of *Trichoderma* isolates against the growth of fungal pathogen *C acutatum*. Plates treated with PDB alone were used as a non-treated control group. The diameter of mycelial growth of fungal pathogens on PDA plates was recorded 7 d after incubation at 25°C. All the experiments were performed twice with triplicates per treatment. Bars with the same letters do not differ from each other according to the least significant difference (LSD) at *P*< 0.05.

We tested the effect of VOCs emitted by *Trichoderma* isolates ATR697 and LON701 against the growth of *C. acutatum* using the overlapping pates assay *in vitro* (**Figure 5A**). The pathogen *C. acutatum* was inhibited by the VOCs produced by ATR697 significantly (*P*< 0.05) to a greater level than the other isolate LON701; while there was no suppression of mycelial growth of *C. acutatum* in a non-treated control. Overall, mycelial growth of the fungal pathogen *C. acutatum* was inhibited to a greater level when a 7-day-old *Trichoderma* isolates culture plate overlapped with *C. acutatum* culture plate than the mycelial growth when *Trichoderma* isolates were inoculated and overlapped on the same day. The mycelial growths were 1.07 and 1.43 cm in the ATR697 and LON701 treatments, respectively, while the mycelial growths were observed as 1.93 and 2.67 cm in ATR697 and LON701 treatments, respectively, when both the pathogen and antagonistic *Trichoderma* isolates were inoculated on the same day.

### Effect of antifungal metabolites secreted by *Trichoderma* isolates against the growth of fungal pathogen *C. acutatum*

We evaluated the ability of *Trichoderma* isolates to secrete metabolites through the cellophane membrane that can suppress the growth of the fungal pathogen *C. acutatum*. When we cultured two *Trichoderma* isolates ATR697 and LON701 on a cellophane membrane overlaid on PDA plates, the mycelial growth of the pathogen was inhibited when cultured onto the site of the cellophane membrane after taking out the cellophane membrane (**Figure 5B**). The growth inhibition rate (%) was found to be significantly (*P*< 0.05) to a greater level in the ATR697 treatment than in the LON701 treatment and the non-treated control. The mycelial growth inhibitions were 5.5 and 6.3 cm in diameter on the ATR697- and LON701-treated plates, respectively. Meanwhile, the inhibition was observed as 8.6 cm in a control, suggesting that the increased mycelial growth inhibition was predominantly from metabolites secreted into the medium.

### *In vitro* antifungal activity by culture filtrates (CFs) of *Trichoderma* isolates

As assessed by *in vitro* antagonistic activities of *Trichoderma* isolates against the fungal pathogen, the ability of the cell-free culture filtrates (CFs) of *Trichoderma* isolates obtained from the two different culture media (PDB and M9) to inhibit the growth of the fungal pathogen, *C. acutatum*, was tested on PDA plates. After 7 d of incubation at 28°C, the colonies were measured for diameter. The colony diameter of the ATR697-CF-treated fungal growth was reduced significantly (*P*< 0.05) to a greater extent than that of the other treatments and the untreated control (**Figure 5C**). The resulting colony diameters were 61.6 and 7.26 cm, in ATR697 and LON701 treatments, respectively, of PDB. Meanwhile, the colony diameters in the ATR697 and LON701 treatments on M9 media were 7.1 and 6.93 cm, respectively. However, there was no significant inhibitory effect of CFs of the two *Trichoderma* isolates from either of the media.

### Control of red pepper anthracnose by *Trichoderma* isolates using curative and preventive methods

**Figure 6:**
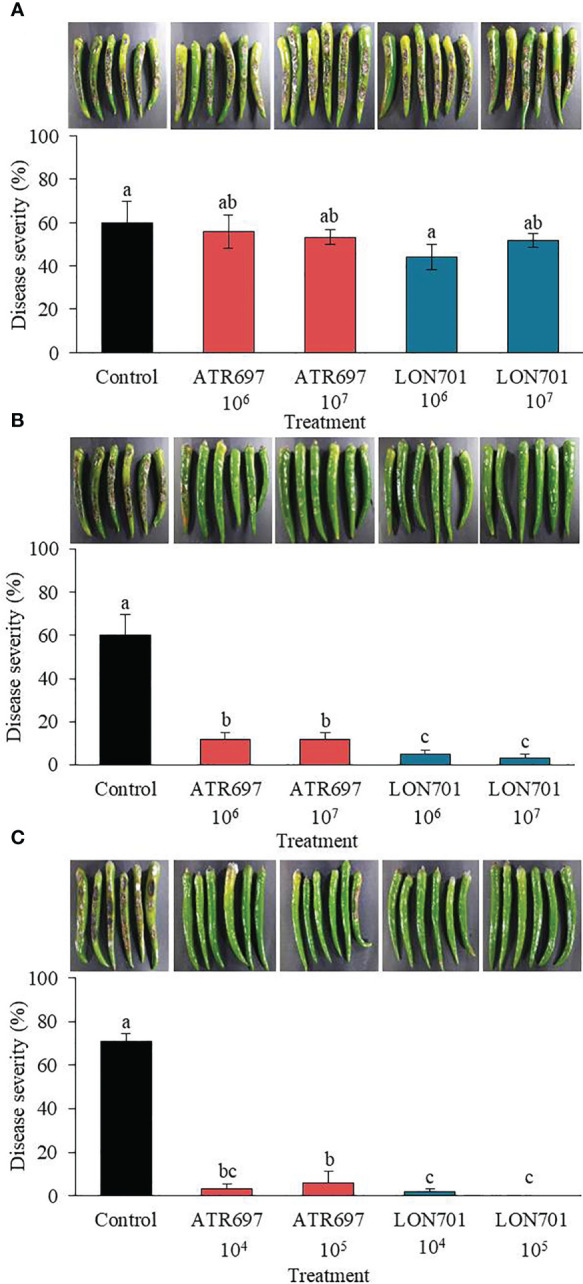
Effect of treatment with *Trichoderma* isolates ATR797 and LON701 on suppression of disease severity (%) of anthracnose caused by *C acutatum* on red pepper fruits by the **(A)** curative method, **(B)** preventive method, and **(C)** preventive method at lower concentrations. Red pepper fruits treated with SDW served as a non-treated control. The disease severity (%) was recorded 7 d after incubation at 25°C. The experiment was performed twice with five replicates. Bars with the same letters do not differ from each other according to the least significant difference (LSD) (*P*< 0.05).

*Trichoderma* isolates, ATR697 and LON701 were tested for their ability to suppress anthracnose disease caused by *C. acutatum* using preventive and curative methods. Red pepper fruits treated with ATR697 or LON701 were inoculated with conidial suspensions of *C. acutatum*. In the curative method, the disease severity (%) was suppressed moderately in both concentrations of *Trichoderma* spore suspensions without any significant differences between *Trichoderma* treatments and the non-treated control (**Figure 6A**). The disease severity in ATR697-treated red pepper fruits was 55.8% and 53.3% at 10^6^ and 10^7^spores/mL, respectively; while the disease severity (%) was 44.1 and 51.6% in the LON701-treated red pepper fruits at 10^6^ and 10^7^spores/mL, respectively. Whereas, the disease severity was 94% in the non-treated control. This result indicates that preventive treatment had the highest disease suppression effect when the *Trichoderma* was inoculated on the wounded red peppers before pathogen inoculation. The percentage of disease severity was suppressed significantly with all treatments and at all concentrations (10^6^ and 10^7^spores/mL) of *Trichoderma* isolates (**Figure 6B**). The disease severity in the preventive method was 11.66% in the ATR697-treated red pepper fruits at both concentrations (10^6^ and 10^7^ spores/mL), while the disease severity was 5% and 3.3% in the LON701-treated red pepper fruits at 10^6^ and 10^7^ spores/mL, respectively. Whereas, non-treated red pepper fruits presented 60% disease severity (%). Based on the effective results of the preventive method, the concentrations of the *Trichoderma* isolates were further reduced to 10^4^ and 10^5^ spores/mL to check their efficacy against pathogen growth. The two *Trichoderma* isolates, ATR697 and LON701, at both concentrations, have displayed the suppression of red pepper anthracnose drastically *ex vivo*, while non-treated control fruits did not exhibit disease suppression (**Figure 6C**). Among all the treatments, there were no diseased lesions in the LON701 treatment at 10^5^ spores/mL. Based on these results, two *Trichoderma* isolates display effective biocontrol activity against red pepper anthracnose.

### Fungicide sensitivity test for *Trichoderma* isolates under *in vitro* conditions

**Figure 7:**
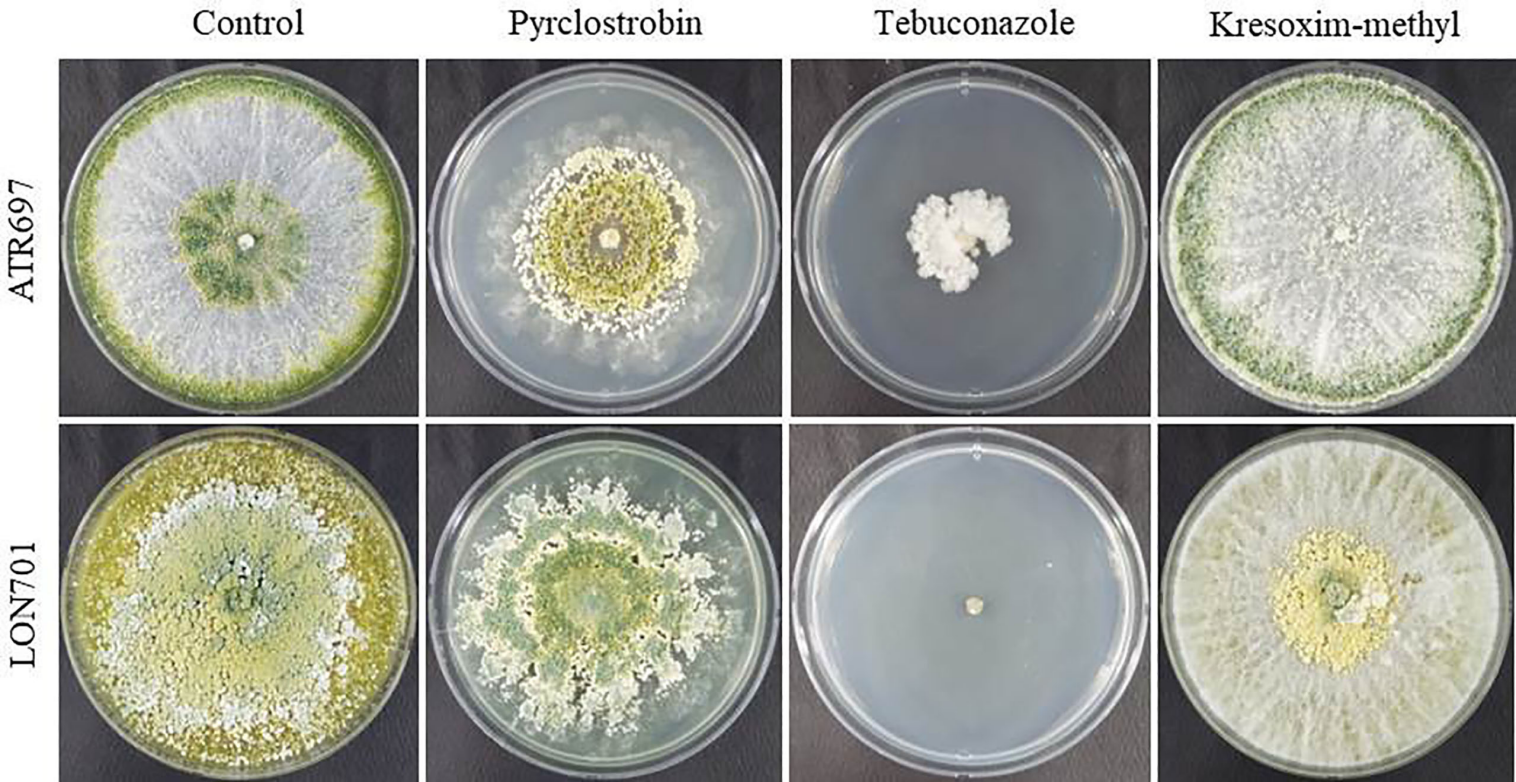
Fungicide sensitivity to *Trichoderma* isolates ATR697 and LON701 under *in vitro* conditions. Mycelial plugs of *Trichoderma* isolates were inoculated onto PDA plates supplemented with fungicides (pyraclostrobin, tebuconazol, and kresoximmethyl). PDA plate without fungicide served as a non-treated control. The mycelial growth was observed 7 d after incubation at 25°C and compared with a non-treated control. The experiment was performed twice in triplicates per treatment. Percentage (%) inhibition of mycelial growth rate was calculated.

The two *Trichoderma* isolates, ATR697 and LON701, were tested for their resistance to various commercial fungicides under *in vitro* conditions. The isolates, ATR697 and LON701 were fully grown on control and kresoxim-methyl-treated PDA plates, while both *Trichoderma* strains were found to be sensitive to tebuconazole (**Figure 7**). However, the LON701 strain was found to be more sensitive than ATR697. The mycelial growth of the ATR697 strain was spread to 50% on a pyraclostrobin-treated plate, while the mycelial growth of the LON701 strain was spread more than 50% of the plate, suggesting the higher resistance to pyraclostrobin than the ATR697 stain.

### Synergistic effect of *Trichoderma* spp. in combination with a commercial fungicide

**Figure 8:**
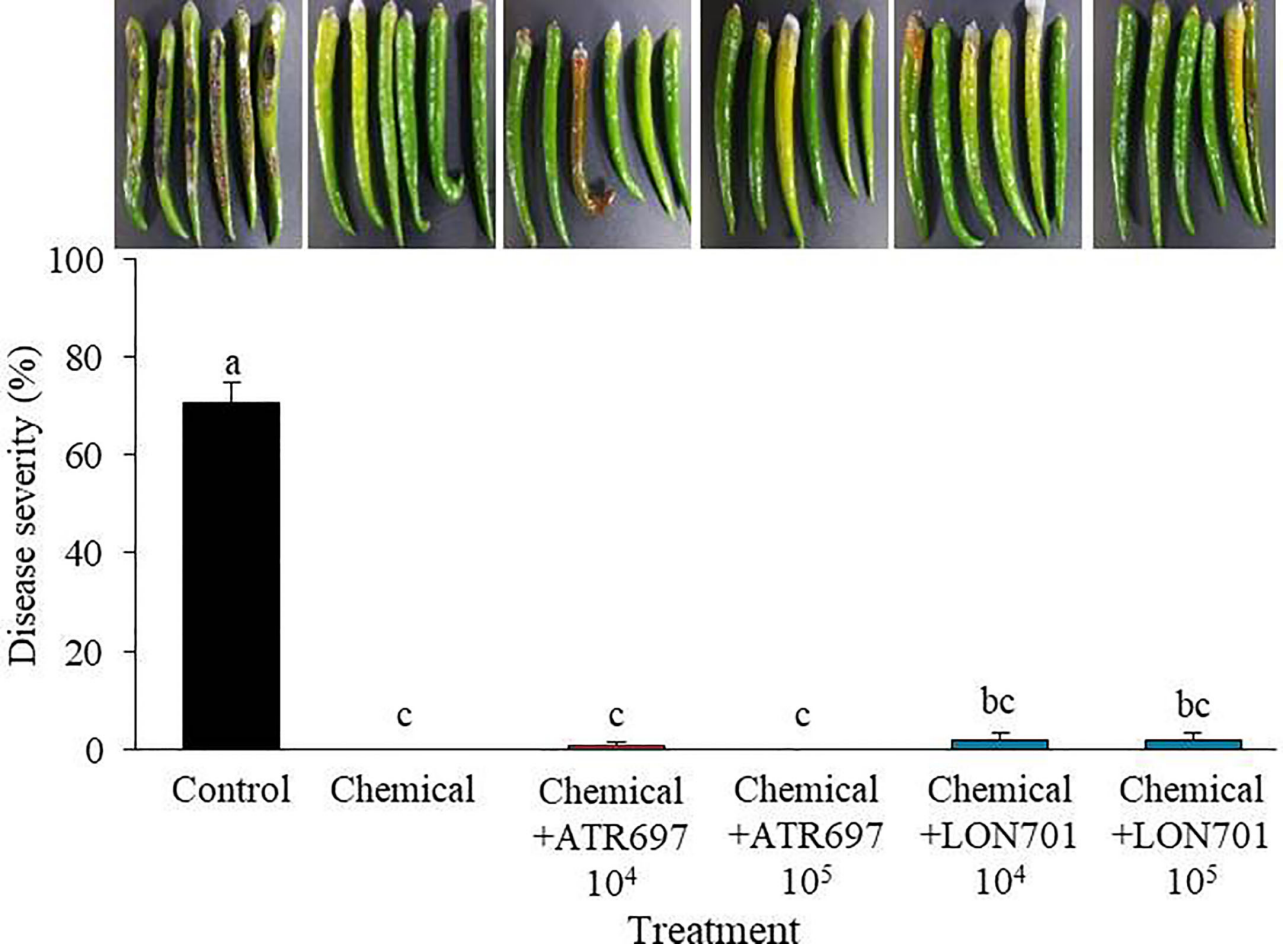
Synergistic effect of spore suspensions of *Trichoderma* isolates in combination with chemical fungicide (pyraclostrobin) on suppression of disease severity (%) of anthracnose caused by *C. acutatum* on red pepper fruits by preventive method at concentrations of 10^4^ and 10^5^ spores/mL. Red pepper fruits treated with SDW served as a non-treated control. The disease severity (%) was recorded 7 d after incubation at 25°C. The experiment was performed twice with five replicates. Bars with the same letters do not differ from each other according to the least significant difference (LSD) (*P*< 0.05).

When *Trichoderma* isolates along with a chemical (pyraclostrobin), are used as treatments to manage red pepper anthracnose *ex vivo*, the four treatments have demonstrated their ability to manage anthracnose disease caused by *C. acutatum* in red pepper fruits using a preventive method (**Figure 8**). The disease severity of anthracnose was reduced to a significant level (*P*< 0.05) when two *Trichoderma* isolates at lower concentrations (10^4^ and 10^5^spores/mL) were used in combination with the chemical fungicide (pyraclostrobin). This finding suggested that the two *Trichoderma* isolates could be used in conjunction with chemical fungicides to boost the effectiveness of biocontrol.

### Effect of *Trichoderma* isolates on red pepper anthracnose disease control in the field

**Figure 9:**
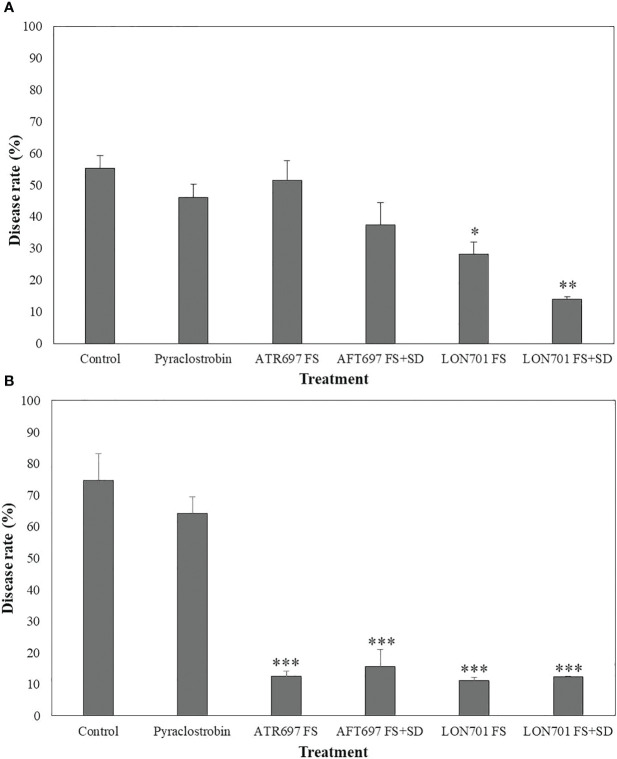
Suppression of anthracnose by treatment with *Trichoderma* isolates ATR697 and LON701 under field conditions in 2021 **(A)** and 2022 **(B)**. After transplanting two-month-old red pepper seedlings in the field, the plants were treated with ATR697 and LON701 spore suspensions by foliar spray or foliar spray + soil drench or water (control), chemical control, and pyraclostrobin (negative control) for seven times in 60 (d) Disease rate (%) was recorded from disease-infected red pepper fruits after 10 d of the last treatment, and compared with chemical and non-treated controls according to the least significant difference (LSD) (***P<0.001, **P<0.01, and *P<0.05). For all the treatments, three different plots with twenty replicates (plants) were used.

Based on *in vitro* and *ex vivo* results of disease suppression of anthracnose by two *Trichoderma* isolates, ATR697 and LON701, these two isolates were investigated for their ability to suppress *C. acutatum*-caused red pepper anthracnose in the field in 2021 and 2022. In 2021, a reduced disease rate (%) was observed in all treatments. However, the treatment with LON701 significantly reduced the red pepper anthracnose infection (*P*< 0.05) by the foliar spray + soil drench method with a disease rate of 14%, when compared to chemical control (pyraclostrobin) and the non-treated control, presented 46.1% and 55.3% of disease rate, respectively (**Figure 9A**). The disease rate was 51.5% and 37.4% by foliar spray and foliar spray + soil drench, respectively, in the ATR697 treatment 10 d after the last treatment. Overall, a significant (*P*< 0.05) reduction of the disease rate (%) was indicated by the LON701 treatment under field conditions, compared to the ATR696 treatment in 2021. However, in 2022, there was no statistically significant difference (*P*< 0.05) between the treatments for two isolates ATR696 and LON701 using foliar spray and foliar spray + soil drench methods for reducing the disease rate of anthracnose. Whereas, the two *Trichoderma* isolates have been found to be more effective in disease suppression, compared to the chemical and the non-treated control with disease rates of 64% and 74.6%, respectively (**Figure 9B**). Between the two years, the disease rate (%) had reduced to some extent in the year 2021 than in 2022, because of changes in weather parameters.

## Discussion

The most destructive disease brought on by *Colletotrichum* spp., anthracnose, particularly in red pepper, has caused a decrease in both quality and output (Ali et al., 2016; Diao et al., 2017). Mostly, farmers rely on conventional chemical pesticides to control various plant diseases, including anthracnose in red peppers (Yadav et al., 2023). However, the continuous application of these chemical pesticides leads to health disorders in human beings and develops resistance to plant pathogens. Therefore, in this study, as an alternative to chemical pesticides, we investigated the antagonistic activity of *Trichoderma* isolates against plant fungal pathogens, including *C. acutatum* which causes anthracnose in red peppers. Fungal growth was inhibited by two antagonistic *Trichoderma* isolates, ATR697 and LON701. Among the three fungal pathogens, *C. acutatum* was strongly suppressed by the two *Trichoderma* isolates. The discovery of different *Trichoderma* species that have been confirmed as BCAs against various fungal phytopathogens has served as additional proof of this (Mazrou et al., 2020). Similar to this, Ruangwong et al. (2021) observed that the strain *Trichoderma koningiopsis* PSU3-2 successfully inhibits the growth of *C. gloeosporioides* through a method of competition. The strong inhibitory effect of *Trichoderma* on phytopathogens has been displayed, including *Botrytis cinerea* (Vos et al., 2015); *Fusarium graminearum* (Saravanakumar et al., 2017) and *Macrophomina phaseolina* (Kouadri et al., 2023) through the production of various volatile and non-volatile secondary metabolites (Saravanakumar and Wang, 2020).

To find out whether the development of *C. acutatum* in the PDA medium is impacted by the VOCs produced by two *Trichoderma* strains, the overlapping plates method was used. We found that VOCs produced by two *Trichoderma* isolates, ATR697 and LON701 significantly inhibited the growth of the mycelial pathogen *C. acutatum* by 83.8% and 78.3%, respectively, 7 d after culture when compared with the non-treated control. Our results are in agreement with those reported previously in other *Trichoderma* species. VOCs produced by several *Trichoderma* species were found to have multiple activities, including antifungal effects, inducing defense response, and promoting plant growth (Phoka et al., 2020). VOCs including azetidine, 2-phenylethanol, and ethyl hexadecanoate, have been reported to exhibit antimicrobial activity (Choi et al., 2010; Angel et al., 2016; Li et al., 2018). As a result, these volatile metabolites were discovered to limit *C. gloeosporioides* mycelial development, implying an antibiosis mechanism (Baiyee et al., 2019). Similarly, several *Trichoderma* species have been reported to secrete certain hydrolytic enzymes, such as chitinase and β-1,3-glucanase responsible for degrading fungal cell walls (Asad et al., 2015).

Cell-free culture filtrates (CFs) obtained from two different media PDB and M9, were used to demonstrate the activity of the CF of two isolates ATR697 and LON701 against the mycelial growth of *C. acutatum*, corresponding to a higher level of growth inhibition activity in CF from PDB than M9 in ATR697. This may be because of various secondary metabolites in the CF of *Trichoderma* isolates. These results are in line with those of Yassin et al. (2022), who showed that CFs of *T. harzianum* and *T. viride* exhibited antifungal potential against various phytopathogenic fungal strains, such as *Alternaria alternata* and *Fusarium proliferatum*, respectively. *Trichoderma* species are known to produce a broad spectrum of non-volatile secondary metabolites, such as trichodermin, trichodermil, fitotripen, and trichozam, which can suppress the growth of a wide range of pathogens (Hyder et al., 2017; Rajani et al., 2021). In addition, several *Trichoderma*-based biopesticides have been made available commercially against various fungal diseases (Błaszczyk et al., 2017; Mulatu et al., 2022). We also investigated whether the ability of secondary metabolites derived from our *Trichoderma* strains to pass through a cellophane membrane is against the mycelial growth of *C. acutatum* on the same Petri dish. Secondary metabolites generated from the two isolates suppressed *C. acutatum* mycelial expansion on PDA plates. However, identifying specific compounds is required to understand the broad range of metabolites in them and those responsible for the growth inhibition of fungal pathogens. The disease severity of anthracnose in the red pepper fruits was controlled by *Trichoderma* isolates using curative and preventive methods. The best disease suppression effect was observed in the preventive treatment in our study. These results support a previous report by Díaz-Gutierrez et al. (2021), where the *T. asperellum* exhibited the best antagonistic effect on the growth of fungal pathogen *F. oxysporum* in the preventive treatment. In our study, the data in a preventive method indicated that the candidate strains, ATR697 and LON701 have shown the ability to control red pepper anthracnose and may show further antagonist effects on other fungal pathogens.

It has been claimed that *Trichoderma* spore suspensions can be used to control a variety of plant diseases (Dawidziuk et al., 2016; Sunpapao et al., 2018). Treatment with spore suspensions of *Trichoderma* isolates, ATR697 and LON701 has been displayed to reduce the severity of red pepper anthracnose caused by *C. acutatum* in this study. Further, understating the sensitivity of the *Trichoderma* isolates to various commercial fungicides may have certain involvements for the effective control of the disease, especially; when BCAs are used in combination with chemical fungicides. In our study, the synergistic effect of *Trichoderma* isolates, ATR697 and LON701 with chemical fungicide (pyroclostrobin) have displayed the lowest/no disease severity (%) in red pepper fruits by the preventive method. These findings are consistent with a recent work by Gonzalez et al. (2020), who found that combining the BCA *T. reesei* C2A with modest doses of mancozeb, a commercially available fungicide, suppressed the mycelial growth of *F. oxysporum in vitro*. Previously, *Trichoderma harzianum* strain T969 (Zamanizadeh et al., 2011) and *Trichoderma viride* (Kari et al., 2012) have been found to exhibit *in vitro* antagonistic activity against several phytopathogens, but not under field conditions. Whereas, in the present study, the two *Trichoderma* isolates, ATR697 and LON701 significantly reduced the red pepper anthracnose disease under field conditions at a greater level when compared to the non-treated control and the chemical control (pyraclostrobin) by the foliar spray and the combination of foliar spray + soil drench. However, in field conditions, disease control of red pepper anthracnose is dependent on the meteorological parameters of each year. Therefore, this field investigation sheds light on how these *Trichoderma* isolates could be used as biocontrol agents to increase their effectiveness and provide long-term protection.

## Conclusions

This work demonstrated the potential of two antagonistic soil-derived *Trichoderma* strains, *T. atroviride* ATR697 and *T. longibraciatum* LON701, as biocontrol agents against *C. acutatum*-caused red pepper anthracnose. These two strains are potential candidates for biological control agents, as they have shown increased inhibitory activity against the growth of fungal pathogens, causing anthracnose in red pepper fruits. The key factor contributing to the suppressive effect of volatile and non-volatile metabolites on the mycelial growth of the fungal pathogen *C. acutatum* that causes red pepper anthracnose is their antifungal activity. *Trichoderma* spp. along with chemical fungicides can synergistically be used for the control of red pepper anthracnose. Our findings suggest that the two strains of *T. atroviride* ATR697 and *T. longibraciatum* LON701 are excellent candidates for the biocontrol of red pepper anthracnose. The mechanism underlying red peppers’ capacity for biocontrol will be clarified by future research.

## Data availability statement

The datasets presented in this study can be found in online repositories. The names of the repository/repositories and accession number(s) can be found in the article.

## Author contributions

SK and YL contributed to design and perform the experiments. SK was involved in the field experiments. YL was involved the molecular identification of the isolate. The manuscript was written by KB and YJ after data analysis. YJ supervised the project. All authors contributed to the article and approved the submitted version.
